# Effect Evaluation of Subxiphoid and Intercostal Thymectomy: A Meta-Analysis and Systematic Review

**DOI:** 10.3389/fsurg.2022.925003

**Published:** 2022-05-31

**Authors:** Hailong Wang, Miao Wang, Ning Xin, Rongqiang Wei, Kenan Huang

**Affiliations:** ^1^ Department of Thoracic Surgery, Suzhou Ninth People's Hospital, Suzhou Ninth Hospital affiliated to Soochow University, Suzhou, China; ^2^ Department of Thoracic Surgery, Shanghai Pulmonary Hospital, School of Medicine, Tongji University, Shanghai, China; ^3^ Department of Thoracic Surgery, Shanghai Changzheng Hospital, Navy Military Medical University, Shanghai, China; ^4^ Department of Thoracic Surgery, The First Affiliated Hospital of Soochow University, Suzhou, China

**Keywords:** systematic review, subxiphoid, intercostal, thymoma, meta-analysis

## Abstract

**Background:**

It still remains unclear whether subxiphoid video-assisted thoracoscopic surgery (SVATS) thymectomy is safe and reasonable. This meta-analysis aims at assessing the effectiveness and safety of SVATS for thymoma in comparison with that of intercostal video-assisted thoracoscopic surgery (IVATS) thymectomy.

**Methods:**

All the relevant data systematically analyzed in this thesis were retrieved from PubMed, the Cochrane Library, web of science, EMBASE, and ClinicalTrials.gov. The time span for data retrieval was from the date of database establishment to March 2022. The outcome indicators include operation time, intraoperative blood loss, duration of postoperative drainage, postoperative hospital days, visual analogue scale (VAS) score on the day of operation, VAS score on postoperative day 3, and VAS score on postoperative day 7; postoperative complications were analyzed in our meta-analysis.

**Results:**

In 13 studies of this paper, there were 1,198 cases included. Among them, 563 cases were treated by SVATS thymectomy and 635 cases by IVATS thymectomy. There was no significant difference in the operation time [113.38 vs. 119.91  min, 95% confidence interval (CI): −0.70–0.15, *p* = 0.20) and the incidence of intraoperative and postoperative complications (RR = 0.82, 95% CI: 0.58–1.15, *p* = 0.25) between SVATS thymectomy and IVATS thymectomy. However, SVATS thymectomy significantly reduced the amount of intraoperative blood loss (47.68 vs. 66.69  mL, SMD = −0.57, 95% CI: −0.95 to −0.18, *p* = 0.004), postoperative drainage days (2.12 vs. 2.72 days, SMD = −0.46, 95% CI: −0.74 to −0.18, *p* = 0.001), postoperative hospital stays (4.53 vs. 5.91 days, SMD = −0.64, 95% CI: −0.96 to −0.31, *p* = 0.0001), and VAS scores after the operation.

**Discussion:**

SVATS thymectomy is safe and feasible, and the perioperative effect is better than IVATS thymectomy to a certain extent, which is worthy of popularization and further research.

**Systematic Review Registration:** https://www.crd.york.ac.uk/PROSPERO/

## Introduction

Thymoma is the most common anterior mediastinal tumor. Surgical resection is still the main treatment for thymoma (1). Transsternal thymectomy is the gold standard for thymoma surgery (2–4). However, with the development of minimally invasive equipment and technology, thoracoscopic thymectomy is gradually favored by thoracic surgeons due to its characteristics of the short operation time, less intraoperative bleeding, and fast postoperative recovery (5–7). The first and most commonly used surgical procedure was lateral thoracoscopic intercostal (IVATS) thymectomy. However, another new surgical approach, thoracoscopic thymectomy via the subxiphoid process (SVATS), has gradually been favored by some thoracic surgeons. However, there is no high-level evidence to confirm the superiority of the two surgical procedures. Therefore, the following meta-analysis and systematic review were conducted to evaluate the advantages and disadvantages of the two surgical methods in thymectomy.

## Methods

This meta-analysis has been registered on the Prospero with number CRD42022321070 and followed the Preferred Reporting Items for Systematic Reviews and Meta-Analyses (PRISMA).

### Surgical Approach

SVATS refers to the complete resection of thymus tissue, tumor, and anterior mediastinal adipose tissue (within the left and right innominate veins and bilateral phrenic nerves) through uniportal or multiportal surgery via the subxiphoid process. IVATS refers to the resection of complete thymus tissue, tumor, and anterior mediastinal adipose tissue as much as possible through uniportal or multiportal surgery via the intercostal process.

### Eligibility Criteria

Inclusion criteria are as follows: (I) It is a study of thoracoscopic thymectomy, (II) randomized controlled trial (RCT) or case-control study or cohort study, and (III) comparison of thymectomy by SVATS and IVATS.

Exclusion criteria are as follows: (I) studies whose language was not English; (II) outcome indicators did not include any of the following: operation time, intraoperative blood loss, duration of postoperative drainage, postoperative hospital days, postoperative VAS score, postoperative complications; (III) reports, conference abstracts, reviews, editorials, or expert opinions; and (IV) research for robot-assisted thoracoscopic surgery.

### Search Strategy and Information Sources

**Figure 1:**
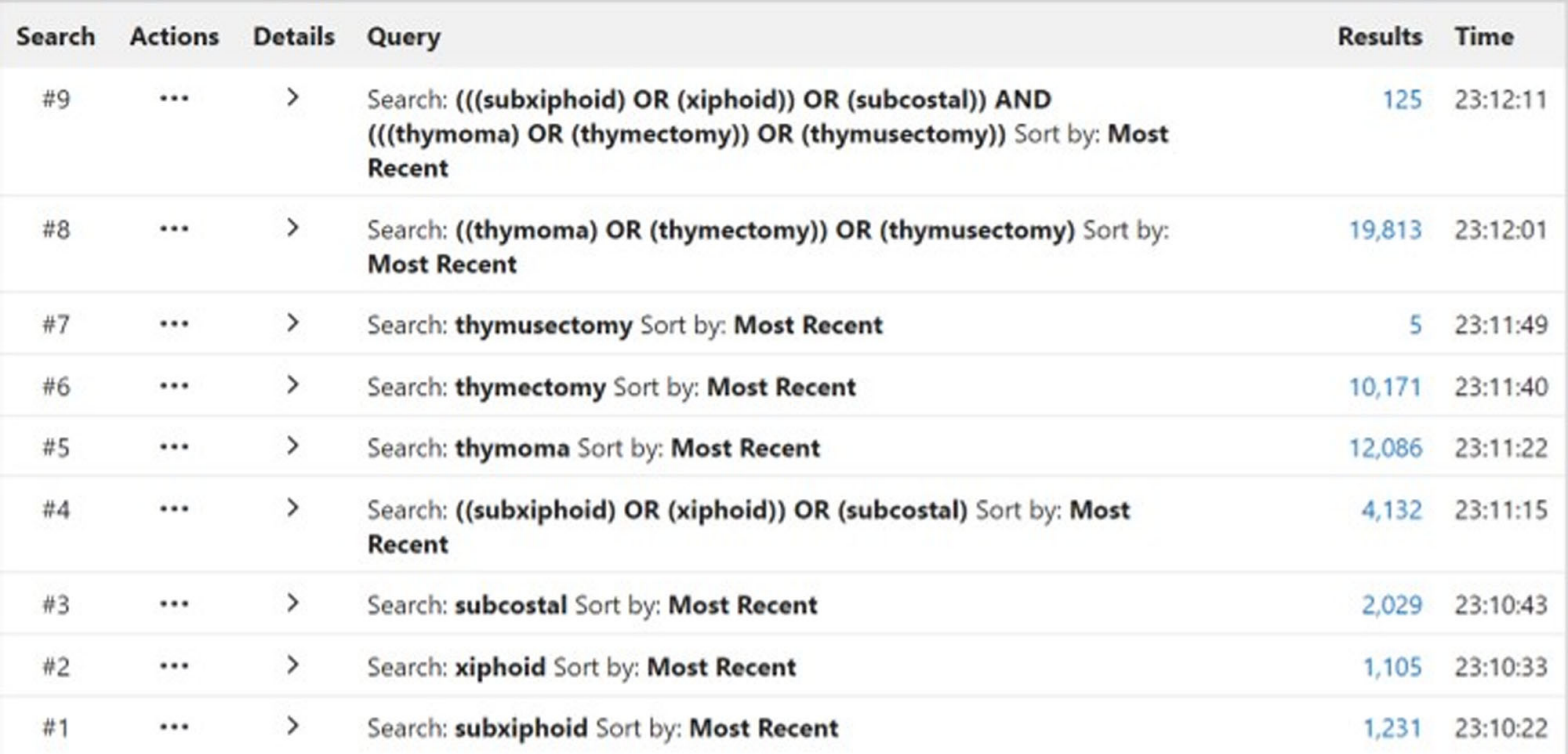
Retrieval strategy.

All literature studies were retrieved from PubMed, the Cochrane Library, web of science, EMBASE, and ClinicalTrials.gov. The time span for data retrieval was from the date of database establishment to March 2022. During the data retrieval process, a combination of subject headings and free words was adopted, and references in the literature review section were retrospectively reviewed to supplement and obtain more relevant information. To facilitate the retrieval of related literature studies, the retrieval strategy we adopted is shown in Figure 1.

### Selection Process

**Figure 2:**
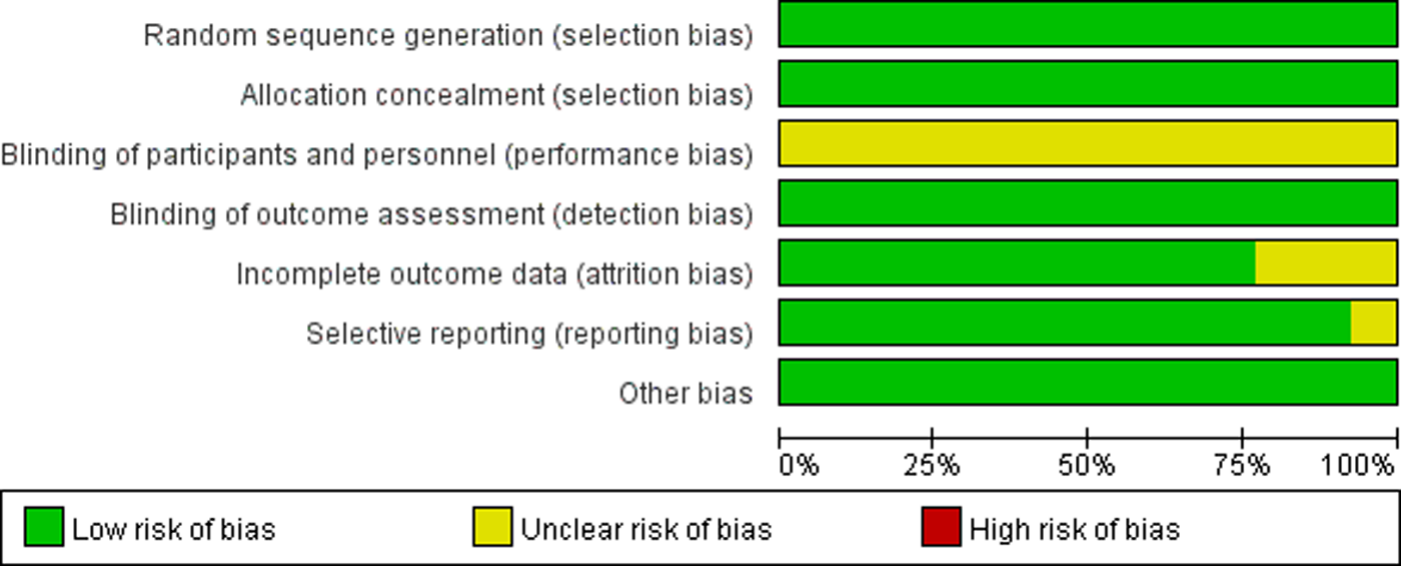
Risk of bias graph according to the Cochrane collaboration’s tool.

**Figure 3:**
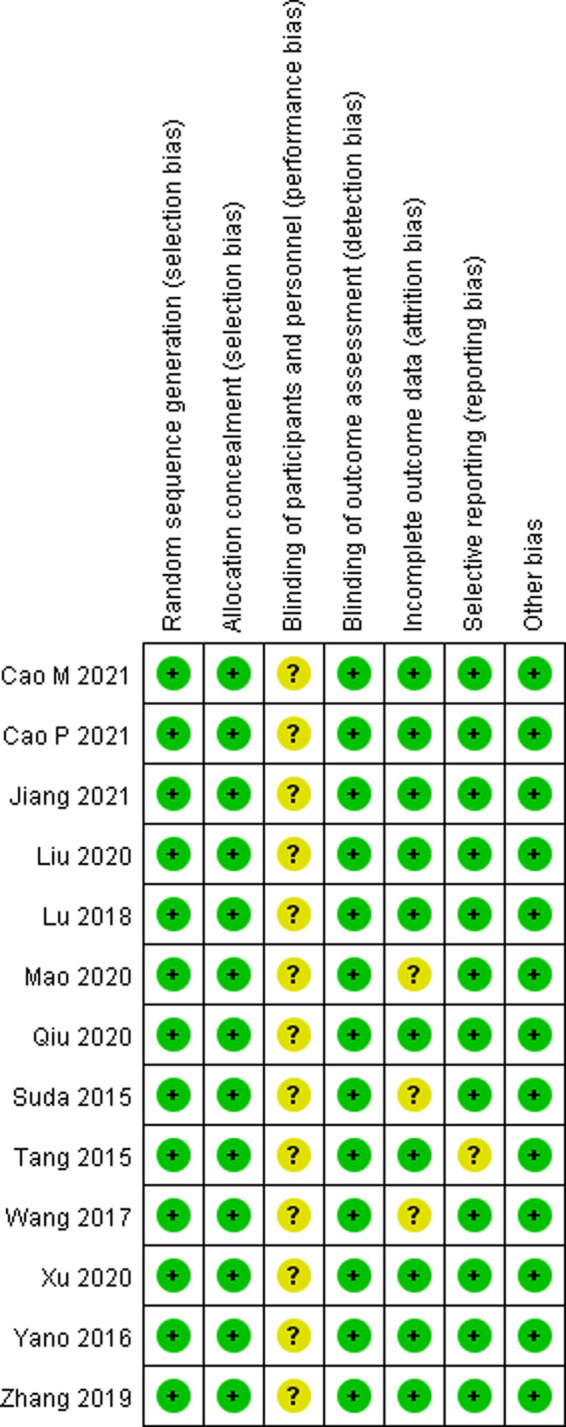
Risk of bias summary according to the Cochrane collaboration’s tool.

Two investigators viewed all the literature studies that we retrieved independently and excluded irrelevant literature studies by scanning the title and abstract. Then, the literature studies that met our meta-analysis were screened by reading the full texts, after downloading them, according to the inclusion and exclusion criteria mentioned previously. In case of disagreement between the two investigators, it was decided by a third investigator after carefully reading the full texts. The risk of bias graph and risk of bias summary are shown in Figures 2, 3.

### Data Extraction

Two investigators read the full text carefully and independently collected data for each report, with the Senior Statistician confirming the study investigators’ data. Outcome indicators collected included some or all of the data described later: operation time, intraoperative blood loss, duration of postoperative drainage, postoperative hospital days, visual analogue scale (VAS) score on the day of operation, VAS score on postoperative day 3, VAS score on postoperative day 7, and postoperative complications. For missing data, we sought from the author of the original study.

### Quality Assessment

The quality of each cohort study was assessed using the Newcastle Ottawa scale (NOS) (8). The NOS in these studies consists of three perspectives with a maximum of nine stars: a maximum of four stars for selection, two stars for comparability, and three stars for the ascertainment of the outcome of interest. Studies with a score of five or more are considered high-quality studies.

### Statistical Analysis

In statistical analysis, relative ratios (RRs) were used for dichotomous variables, and mean differences (MDs) or normalized mean differences (SMDs) were used for continuous variables with 95% confidence intervals (CI). According to the summarized statistical data, *p* value <0.05 indicates that the results are statistically significant. With regard to the literature only with the median and upper and lower limits, “mean variance estimation” was used to estimate the mean and standard deviation according to Luo et al. (9). We used the *χ*^2^-based *Q*-statistic test and *I*^2^ test to evaluate statistical heterogeneity. *I*^2^ < 50% and *p* value >0.10 meant acceptable heterogeneity, and then, the fixed effect model was applied. In contrast, if there was significant heterogeneity, the random effect model was used. Review Manager V.5.3 was used to conduct all statistical analyses.

## Results

**Figure 4:**
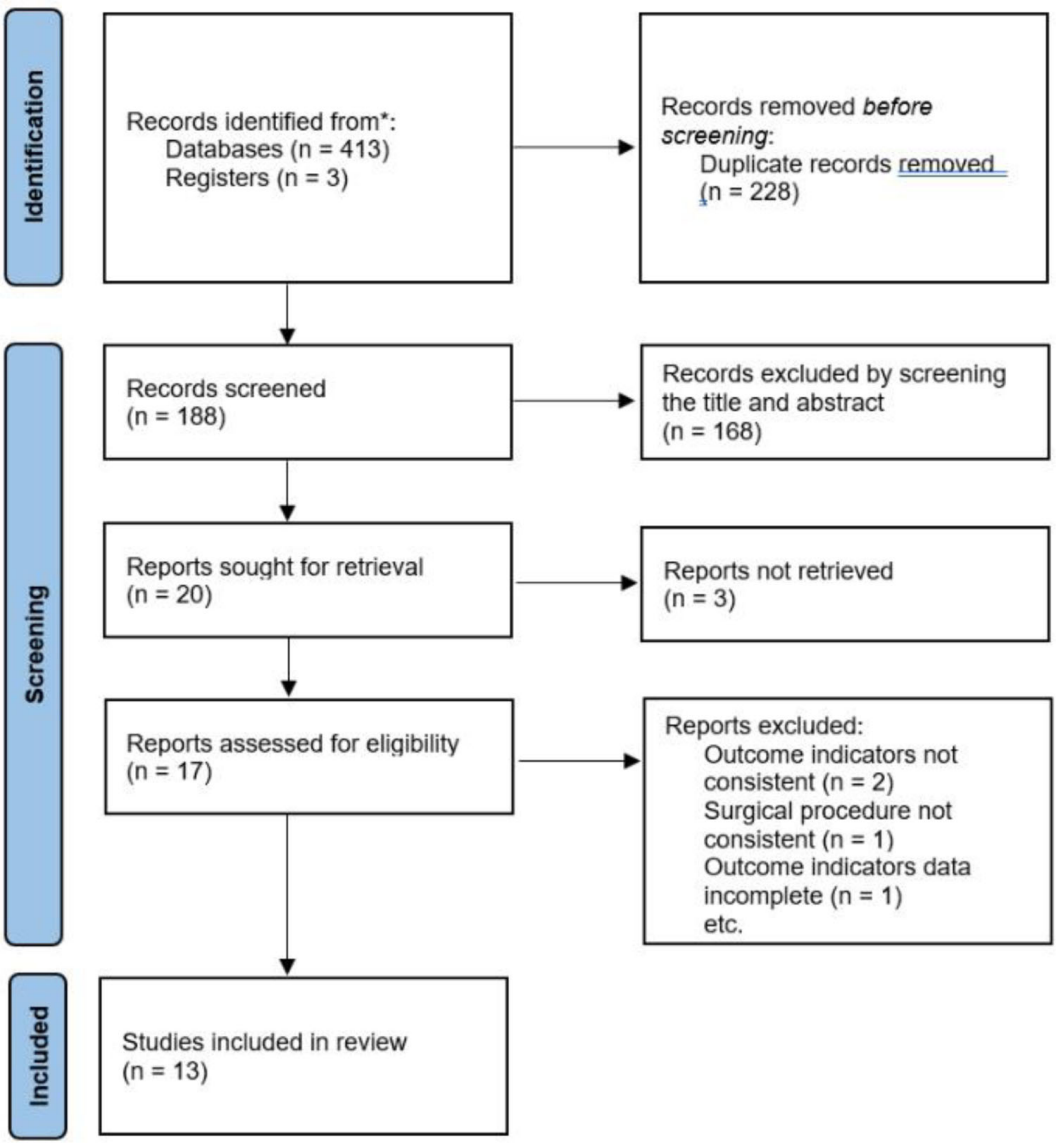
Literature screening process.

According to the retrieval strategy shown in Figure 1, we retrieved 416 relevant studies, including PubMed 125, EMBASE 141, The Cochrane Library 7, web of science 143, and ClinicalTrials Gov 3 items. A total of 228 duplicate studies were excluded, and 168 articles not related to this meta-analysis were eliminated by scanning the title and abstract. After downloading the full text of the remaining 20 literature studies and reading them carefully, one outcome indicator was inconsistent with this study, one operation method was inconsistent with this study, two outcome indicators were incomplete, and three did not find the full text. The above seven literature studies were excluded. We eventually included 13 literature studies with 1,198 cases. Among them, 563 cases were treated by SVATS thymectomy and 635 cases by IVATS thymectomy. The literature screening process is shown in Figure 4. Baseline information and quality assessment of the 13 studies are shown in Tables 1, 2.

**Table 1:** Baseline information of included articles.

First author	Publication year	Ethnicity	Number of patients (SVATS/IVATS)	Age (SVATS/IVATS)	Quality assessment
Suda (18)	2015	Japan	46/35	53.9 ± 14.4/49.7 ± 17.8	NOS 6
Tang (30)	2015	China	20/25	NS	NOS 6
Yano (23)	2016	Japan	14/46	56.5 ± 16.7/48.2 ± 17.9	NOS 5
Wang (31)	2017	China	36/47	NS	NOS 6
Lu (32)	2018	China	41/36	36.3 ± 8.2/38.5 ± 9.1	NOS 6
Zhang (26)	2019	China	28/28	56.2 ± 9.5/58.2 ± 10.0	NOS 6
Xu (22)	2020	China	37/70	53.1 ± 12.0/49.1 ± 13.6	NOS 6
Qiu (28)	2020	China	68/63	45.2 ± 16.8/39.4 ± 17.2	NOS 6
Mao (24)	2020	China	39/39	48.3 ± 14.3/42.9 ± 16.7	NOS 6
Liu (33)	2020	China	76/76	49.8 ± 9.2/ 50.9 ± 9.5	NOS 6
Jiang (34)	2021	China	39/39	48.0 ± 14.0/52.0 ± 15.0	NOS 6
Cao (35)	2021	China	65/72	48.0 ± 10.5/50.9 ± 11.1	NOS 6
Cao (36)	2021	China	57/51	56.8 ± 18.5/52.5 ± 11.7	NOS 6

*IVATS, intercostal video-assisted thoracoscopic surgery; SVATS, subxiphoid video-assisted thoracoscopic surgery; NS, not significant*.

**Table 2:** Baseline information of included articles (SVATS/IVATS).

First Author	Age	Number of patients	Male	BMI	Size of the tumor (cm)	Stage of thymoma		MG patients	Comorbidity
						SVATS (I/II)	IVATS (I/II)		
Suda (18)	53.9 ± 14.4/49.7 ± 17.8	46/35	23/15	23.1 ± 2.9/21.8 ± 3.6	2.92 ± 1.66/3.09 ± 1.63	7/4	6/2	11/10	NA
Yano (23)	48.2 ± 17.9/56.5 ± 16.7	14/46	7/26	NA	4.50 ± 2.70/4.30 ± 3.30	NA	NA	NA	NA
Lu (32)	36.3 ± 8.2/38.5 ± 9.1	41/36	13/16	NA	NA	NA	NA	NA	NA
Zhang (26)	58.2 ± 10.0/56.2 ± 9.5	28/28	16/11	22.7 ± 3.8/24.1 ± 4.3	3.20 ± 1.60/3.40 ± 1.60	14/14	10/18	0/0	NA
Xu (22)	53.1 ± 12.0/49.1 ± 13.6	37/70	19/32	NA	3.73 ± 2.25/3.50 ± 2.22	3/6	10/19	16/28	11/17
Qiu (28)	45.2 ± 16.8/39.4 ± 17.2	68/63	38/38	NA	2.87 ± 1.12/3.14 ± 0.98	24/38	32/30	NA	NA
Mao (24)	48.3 ± 14.3/42.9 ± 16.7	39/39	21/20	23.2 ± 3.2/23.4 ± 3.4	7.10 ± 3.00/5.90 ± 2.40	NA	NA	NA	9/11
Liu (33)	50.9 ± 9.5/ 49.8 ± 9.2	76/76	43/42	23.1 ± 2.9/ 23.6 ± 3.1	3.40 ± 1.70/3.02 ± 1.80	31/43	32/41	NA	12/12
Jiang (34)	48.0 ± 14.0/ 52.0 ± 15.0	39/39	19/20	22.8 ± 3.1/23.1 ± 3.1	6.90 ± 2.60/6.70 ± 2.90	23/16	22/17	NA	9/8
Cao (35)	48.0 ± 10.5/50.9 ± 11.1	65/72	31/37	22.7 ± 3.0/22.4 ± 2.9	3.41 ± 1.38/ 3.27 ± 1.49	15/4	18/4	NA	25/32
Cao (36)	56.8 ± 18.5/52.5 ± 11.7	57/51	13/19	23.2 ± 2.5/22.3 ± 2.4	3.60 ± 2.26/ 3.64 ± 2.45	8/29	11/22	7/3	32/27

*BMI, body mass index; IVATS, intercostal video-assisted thoracoscopic surgery; NA, not applicable; SVATS, subxiphoid video-assisted thoracoscopic surgery; MG, myasthenia gravis*.

**Figure 5:**
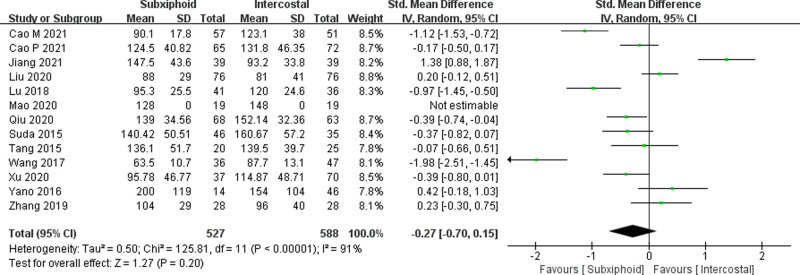
Forest plot of the role of SMD and its 95% CI of operation time between SVATS and IVATS groups.

**Figure 6:**
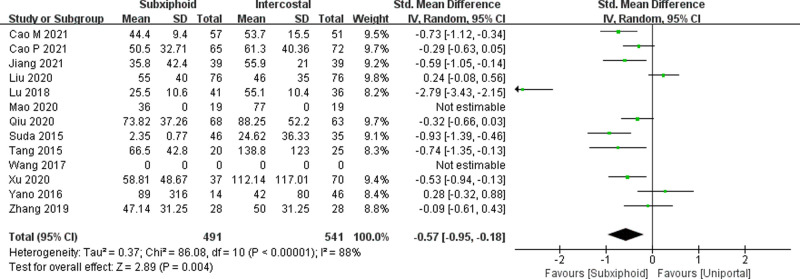
Forest plot of the role of SMD and its 95% CI of blood loss between SVATS and IVATS groups.

**Figure 7:**
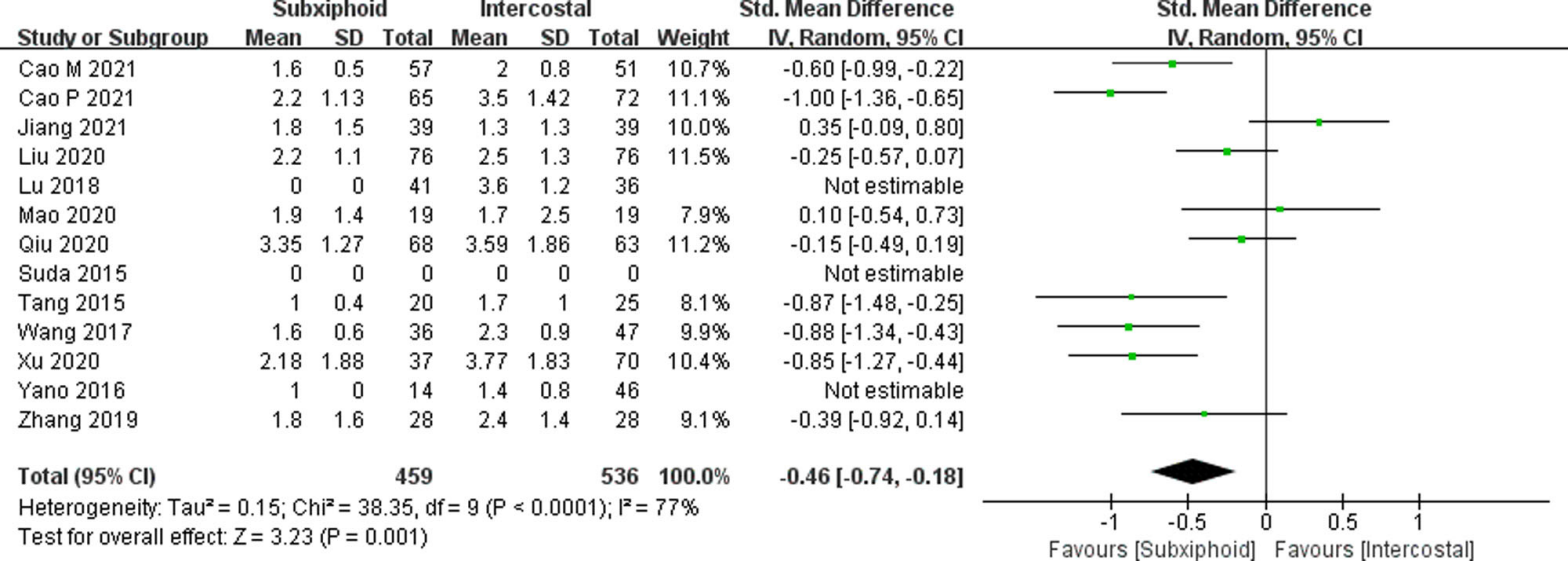
Forest plot of the role of SMD and its 95% CI of postoperative drainage days between SVATS and IVATS groups.

**Figure 8:**
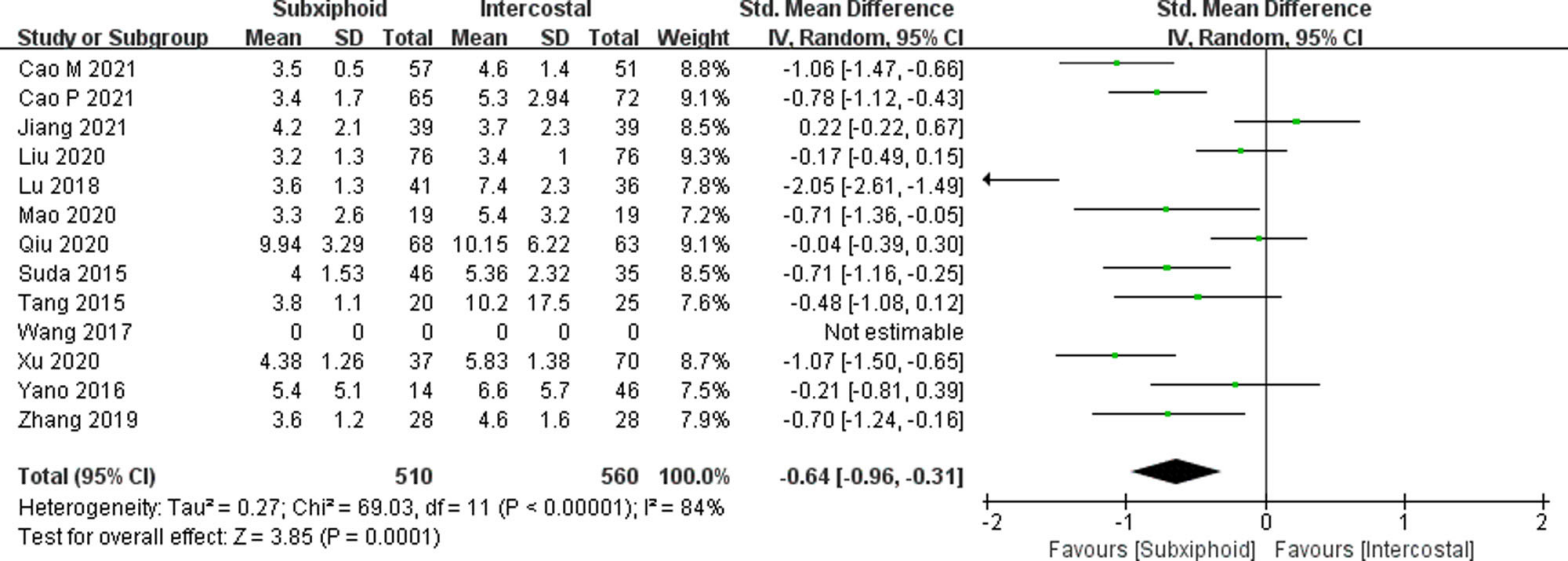
Forest plot of the role of SMD and its 95% CI of postoperative hospital stays between SVATS and IVATS groups.

**Figure 9:**
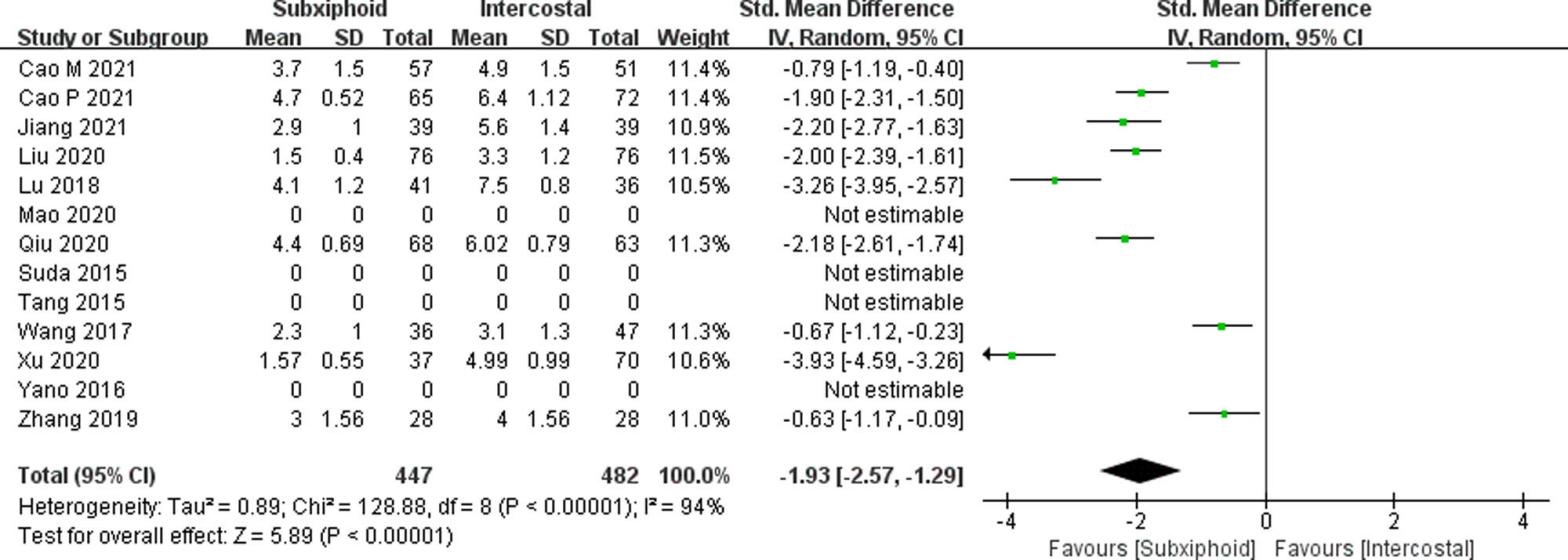
Forest plot of the role of SMD and its 95% CI of VAS score on the day of operation between SVATS and IVATS groups.

**Figure 10:**
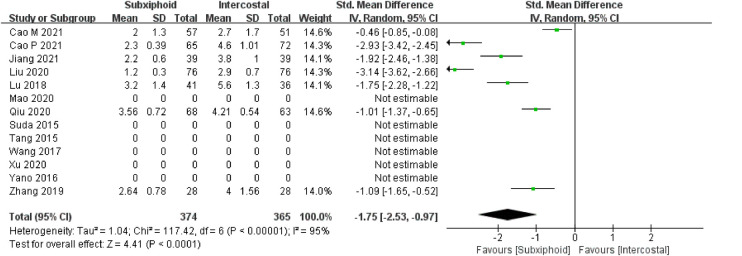
Forest plot of the role of SMD and its 95% CI of VAS score on the third day postoperation between SVATS and IVATS groups.

**Figure 11:**
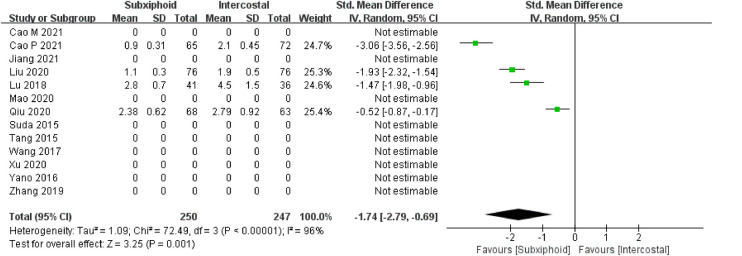
Forest plot of the role of SMD and its 95% CI of VAS score on the seventh day postoperation between SVATS and IVATS groups.

**Figure 12:**
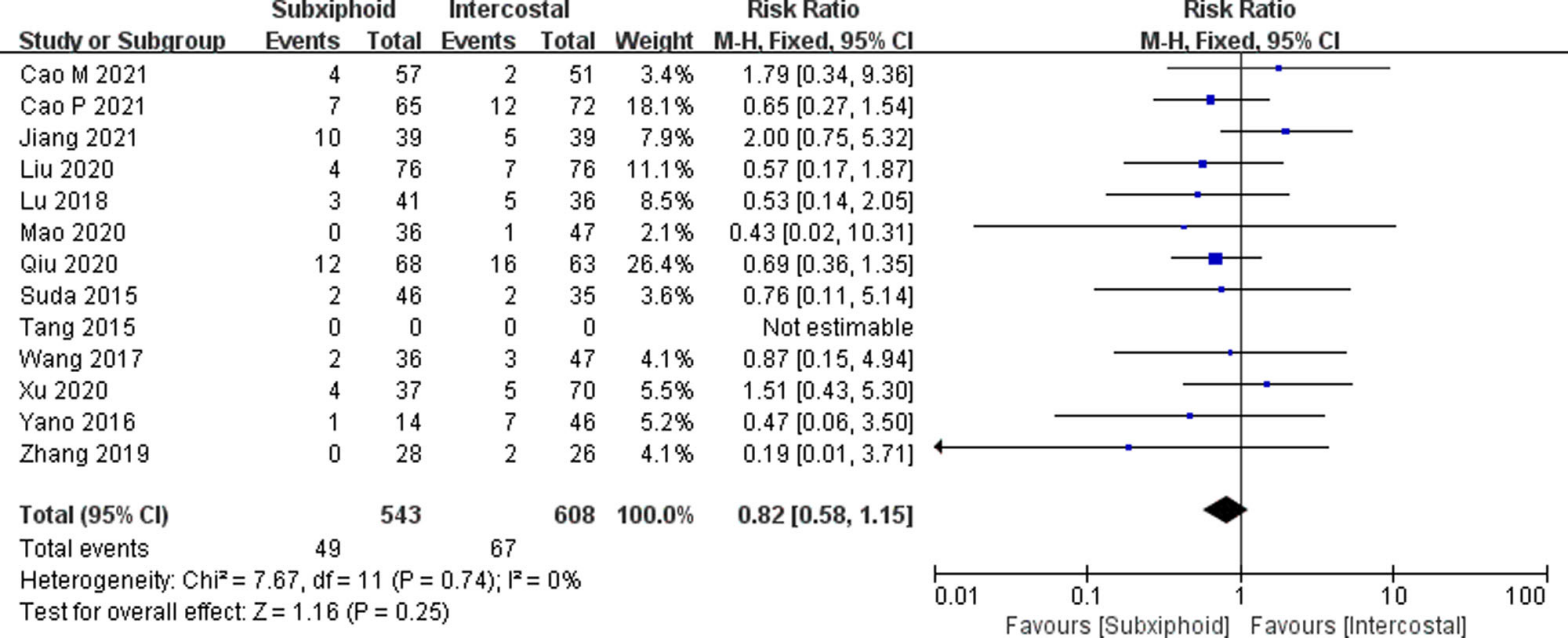
Forest plot of the role of RR and its 95% CI of interoperative and postoperative complications between SVATS and IVATS groups.

We found that there was no statistically significant difference in the average operation time between the two groups, SVATS vs. IVATS (113.38 vs. 119.91  min, SMD = −0.27, 95% confidence interval: −0.70 to 0.15, *p* = 0.20), as shown in Figure 5. The amount of intraoperative blood loss in the SVATS group was significantly less than that in the IVATS group (47.68 vs. 66.69  mL, SMD = −0.57, 95% confidence interval: −0.95 to −0.18, *p* = 0.004), as shown in Figure 6. The postoperative drainage days in the SVATS group were significantly less than those in the IVATS group (2.12 vs. 2.72 days, SMD = −0.46, 95% confidence interval: −0.74 to −0.18, *p* = 0.001), as shown in Figure 7. The postoperative hospital stay in the SVATS group was significantly shorter than that in the IVATS group (4.53 vs. 5.91 days, SMD = −0.64, 95% confidence interval: −0.96 to −0.31, *p* = 0.0001), as shown in Figure 8. The VAS score of the SVAT group was significantly lower than that of the IVATS group on the day after the operation (3.21 vs. 5.05, SMD = −1.93, 95% confidence interval: −2.57 to −1.29, *p* < 0.00001), as shown in Figure 9. The VAS score of the SVAT group was significantly lower than that of the IVATS group on the third day after the operation (2.37 vs. 3.88, SMD = −1.75, 95% confidence interval: −2.53 to −0.97, *p* < 0.0001), as shown in Figure 10. The VAS score of the SVAT group was significantly lower than that of the IVATS group on the seventh day after the operation (1.67 vs. 2.56, SMD = −1.74, 95% confidence interval: −2.79 to −0.69, *p* = 0.001), as shown in Figure 11. There was no significant difference in the incidence of intraoperative and postoperative complications between the two groups (RR = 0.82, 95% confidence interval: 0.58–1.15, *p* = 0.25), as shown in Figure 12.

## Discussion

Thymoma is the most common anterior mediastinal tumor. Surgical resection is still one of the main methods for the treatment of thymoma. Masaoka (10) showed that due to the high incidence of gross anatomical variation of the thymus, ectopic thymus tissue may extend to the esophagus and diaphragm as a widely distributed focus in mediastinal fat. Jaretzki and Wolff (11) also pointed out that the incidence of the ectopic thymus in the neck and mediastinal fat is 98%, so it needs to be removed at the same time. As the ectopic thymic tissue is widely distributed in the anterior mediastinum (12), the International Thymic Malignancy Interest Group pointed out that thymectomy should be a resection of the tumor, thymus, and anterior mediastinal fat tissue completely (13). The integrity resection of the above tissue is the most important factor to determine the long-term survival of patients with thymoma (14–16). Since Blalock proposed transsternal thymectomy in 1939 (17), it has always been the gold standard for surgical treatment of thymoma (4). With the continuous development of endoscopic technology, thoracoscopic thymectomy has become the main method of thymectomy because of its short operation time, less intraoperative blood loss, and fast postoperative recovery. Intercoastal thoracoscopic thymectomy is the most commonly used surgical method at present (6). However, it is difficult to expose the contralateral phrenic nerve and the anterior mediastinum through the intercostal approach, so it may cause the residual thymus tissue and become a potential risk of recurrence in the future (18). Otherwise, it is also inconvenient to expose the upper pole of the thymus through intercostals (18, 19), which may cause damage to the brachiocephalic vein. Therefore, some thoracic surgeons choose bilateral intercostal thoracoscopic thymectomy. However, the injury of the intercostal nerve through the intercostal approach will cause postoperative chest pain and numbness (20, 21). Kido (13) first reported SVATS thymectomy in 1999 and pointed out that SVAT thymectomy can solve the above problems. After that, the subxiphoid approach has increasingly become the preferred surgical method for thoracic surgeons.

Our analysis showed that there was no significant difference in the operation time and the incidence of intraoperative and postoperative complications between SVATS thymectomy and IVATS thymectomy. However, SVATS thymectomy significantly reduced the amount of intraoperative blood loss, postoperative drainage days, and postoperative hospital stays. We think that the decrease in intraoperative blood loss is mainly due to the better exposure to the surgical field of vision, including tumor, thymus tissue, and mediastinal fat tissue through the subxiphoid approach. On one hand, it reduces the possibility of accidental injury. On the other hand, a better surgical field of vision can fully display the small blood vessels such as the thymus vein, so that the surgeon can deal with them more accurately. At the same time, a more accurate intraoperative operation is bound to reduce the days of postoperative drainage to reduce the days of postoperative hospital stay. Therefore, the hospitalization cost is reduced accordingly (18, 22, 23). More importantly, our study found that SVATS thymectomy significantly reduced the VAS scores of patients on the day, the third day and seventh day after the operation. However, whether uniportal or multiportal surgery, the IVATS approach will cause damage to the intercostal nerve to varying degrees (24), causing postoperative long-term chest pain and numbness. The SVATS can avoid the injury to the intercostal nerve and reduce the postoperative pain to a great extent, especially within one week after operation (18, 22, 23, 25). Meanwhile, Zhang et al. (26) pointed out that the incision cosmetic score of SVATS thymectomy is significantly higher than that of IVATS. Otherwise, Masaoka et al. (10) also suggested that the patients in the flat position and single lumen endotracheal intubation in SVTAS thymectomy have less damage to the respiratory system and are conducive to postoperative recovery.

Therefore, without increasing the operation time and complications incidence, SVATS can well reduce the amount of intraoperative blood loss, postoperative drainage time, and postoperative hospital stay and greatly reduce the postoperative pain of patients. At the same time, SVATS thymectomy can not only provide better surgical vision but also help thoracic surgeons to perform the complete resection of the tumor, thymus tissue, and anterior mediastinal adipose tissue more easily (27). So, we believe that SVATS is more suitable for thymectomy than IVATS. However, Mao et al. (24) and Qiu et al. (28) also pointed out that it should be used with caution for obese patients [body mass index (BMI) greater than 30] and patients with poor cardiac function in SVTAS thymectomy. Because the operation space of the SVATS is small, the excessive mediastinal fat in obese patients may further reduce the operation space and increase the intraoperative risk. In addition, the SVATS may squeeze the heart to a certain extent, so it should also be used with caution for patients with poor cardiac function to avoid the occurrence of cardiovascular accidents. At the same time, the pathologist of our group found that only two patients with Masaoka stage III were included in Liu’s (29) research, while the others were stages I and II in all the included studies. We believe that clinicians tend to choose a transsternal approach for patients with thymoma invading adjacent tissues or organs, after weighing the effect and safety of surgery. Therefore, we speculate that clinicians are more interested in choosing the transsternal approach rather than IVATS or SVATS for these Masaoka III patients, so it is unsuitable to compare the IVATS and SVATS approach for patients with Masaoka stage III.

Our research has some limitations. The studies we included are basically retrospective studies without RCT, so there are some limitations in the level of evidence and credibility. In addition, there is no data related to patient survival analysis in the study we included, so the evaluation of the therapeutic effect of SVATS thymectomy on thymoma needs to be further studied. Third, because the SVATS thymectomy is still a relatively novel surgical method, and the surgical proficiency of different surgeons is different, it may have a certain influence on the relevant research results in the perioperative period.

## Conclusions

Our meta-analysis shows that SVATS thymectomy is safe and feasible, and the perioperative effect is better than IVATS thymectomy to a certain extent, which is worthy of popularization and further research.

## Data Availability

The original contributions presented in the study are included in the article/Supplementary Material; further inquiries can be directed to the corresponding author/s.
